# Inference of an Integrative, Executable Network for Rheumatoid Arthritis Combining Data-Driven Machine Learning Approaches and a State-of-the-Art Mechanistic Disease Map

**DOI:** 10.3390/jpm11080785

**Published:** 2021-08-12

**Authors:** Quentin Miagoux, Vidisha Singh, Dereck de Mézquita, Valerie Chaudru, Mohamed Elati, Elisabeth Petit-Teixeira, Anna Niarakis

**Affiliations:** 1 Université Paris-Saclay, Univ Evry, Laboratoire Européen de Recherche pour la Polyarthrite rhumatoïde-Genhotel, 91057 Evry, France; quentin.miagoux@univ-evry.fr (Q.M.); vidisha.kumar@univ-evry.fr (V.S.); dereckdemezquita@gmail.com (D.d.M.); valerie.chaudru@univ-evry.fr (V.C.); elisabeth.teixeira@univ-evry.fr (E.P.-T.); 2 CANTHER, University of Lille, CNRS UMR 1277, Inserm U9020, 59045 Lille, France; mohamed.elati@univ-lille.fr; 3 Lifeware Group, Inria, Saclay-île de France, 91120 Palaiseau, France

**Keywords:** network inference, integrative biology, rheumatoid arthritis, signaling cascades, gene regulation, transcription factors, Boolean simulations, systems biology

## Abstract

Rheumatoid arthritis (RA) is a multifactorial, complex autoimmune disease that involves various genetic, environmental, and epigenetic factors. Systems biology approaches provide the means to study complex diseases by integrating different layers of biological information. Combining multiple data types can help compensate for missing or conflicting information and limit the possibility of false positives. In this work, we aim to unravel mechanisms governing the regulation of key transcription factors in RA and derive patient-specific models to gain more insights into the disease heterogeneity and the response to treatment. We first use publicly available transcriptomic datasets (peripheral blood) relative to RA and machine learning to create an RA-specific transcription factor (TF) co-regulatory network. The TF cooperativity network is subsequently enriched in signalling cascades and upstream regulators using a state-of-the-art, RA-specific molecular map. Then, the integrative network is used as a template to analyse patients’ data regarding their response to anti-TNF treatment and identify master regulators and upstream cascades affected by the treatment. Finally, we use the Boolean formalism to simulate *in silico* subparts of the integrated network and identify combinations and conditions that can switch on or off the identified TFs, mimicking the effects of single and combined perturbations.

## 1. Introduction

Rheumatoid arthritis (RA) is an inflammatory, autoimmune disease that affects the joints of the body. While the exact aetiology is unknown, it involves a combination of environmental and genetic factors such as smoking and susceptibility genes, along with sex and age factors. RA affects 0.5–1% of the world population, with women three times more susceptible to developing RA than men [1,2]. The onset of the disease is set around the fourth to fifth decade of one’s life [3] and, if left untreated, it can be debilitating for the individual. Symptoms of RA include synovial inflammation, joint stiffness and pain, cartilage destruction, and bone erosion. In early RA, leukocytes invade the synovial joints, followed by other pro-inflammatory mediators, instigating an inflammatory cascade and provoking synovitis [2]. In addition, activated monocytes and T cells, both a source of pro-inflammatory cytokines such as TNF-a, can be found in peripheral blood [4], and many RA studies have used peripheral blood cells to identify disease-related genes [5,6,7,8].

The typical therapy for RA includes the use of disease-modifying anti-rheumatic drugs (DMARDs). Conventional DMARDs include drugs that target the entire immune system, whereas biologic DMARDs are monoclonal antibodies (mAbs) and soluble receptors that target protein messenger molecules or cells. Patients who do not respond to conventional DMARDs usually initiate therapy with TNF inhibitors. However, approximately 30–40% of RA patients fail to respond to anti-TNF therapy and are usually obliged to undergo several rounds of drug combinations [9]. Due to the complex nature of RA, systems biology and integrative approaches are needed to gain insight into the disease pathogenesis and progression. In addition, focusing only on one aspect of the disease provides a limited understanding of the multifactorial nature of RA.

Recently, many computational approaches, mainly network-based, which rely on integrating multi-omics data (proteomics, genomics, transcriptomics, and metabolomics), have succeeded in unravelling key mechanisms in complex diseases [10,11,12,13]. In this direction, machine learning is a promising bioinformatics field that allows the use and integration of various biomedical data with inherent complexity and large size. Furthermore, studies have shown that incorporating prior knowledge to data-driven methodologies improves the quality and the biological relevance of the outcome [14,15,16]. One such machine learning tool is CoRegNet, which is an R/Bioconductor package that infers co-regulatory networks of transcription factors (TFs) and target genes by analysing transcriptomic data and estimating TFs activity profiles. Moreover, the software also allows for network enrichment by integrating regulation evidence for TF binding sites, protein–protein interaction data, and chromatin immunoprecipitation (ChIP) data from various databases to support cooperative TFs [17]. In this work, we present a framework for integrating signalling and transcriptional regulation cascades with genomic mutations, combining data-driven approaches with prior knowledge in the form of an integrative RA-specific network. To do so, we use publicly available transcriptomic data of white blood cells from patients suffering from RA and the tool CoRegNet to infer a co-regulatory network.

**Figure 1:**
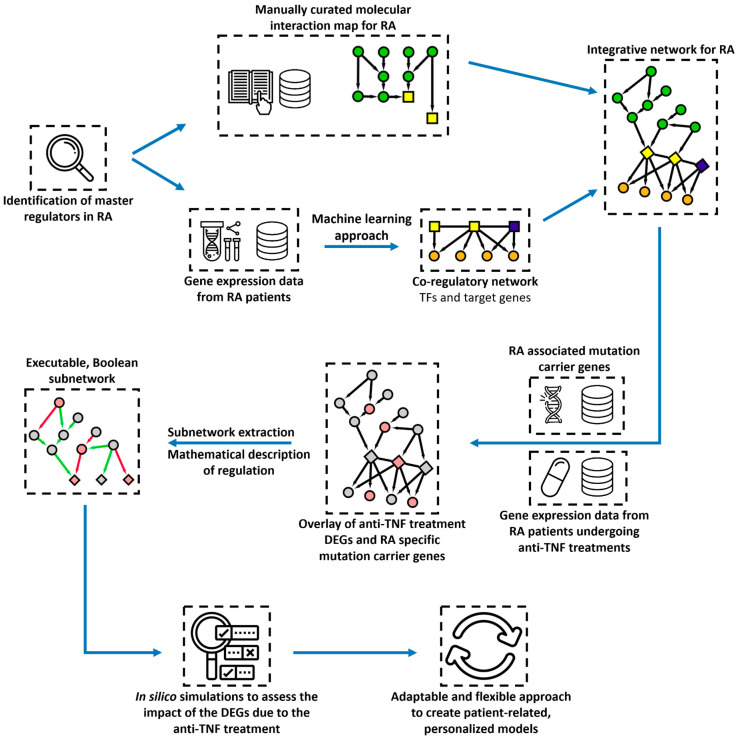
Workflow for creating an integrative and executable network for rheumatoid arthritis (RA). The steps include network inference and identification of master regulators, the combination of the co-regulatory network with curated signalling cascades, the analysis of omics data of anti-TNF treatment, and the addition of Boolean rules to create adaptable and flexible personalised models for *in silico* simulations.

Next, we develop an integration pairing method to couple the RA co-regulatory network with a state-of-the-art disease map for RA [18] to enrich the cooperativity network with upstream signalling regulators. Disease maps are comprehensive, knowledge-based representations of disease mechanisms, including disease-related molecular interactions supported by literature-based evidence [19,20]. Next, we project on the integrative RA network public genomic data and transcriptomic data from treated RA patients, highlighting key mutation carriers and differentially expressed genes associated with the response to anti-TNF treatment (Figure 1). The goal is to unravel mechanisms governing the regulation of key transcription factors and genes identified as mutation carriers or DEGs in RA patients undergoing anti-TNF treatment.

Lastly, we study the system’s dynamic behaviour using Boolean formalism to simulate subparts of the integrated network [21,22]. We perform real-time simulations, sensitivity analysis, and dose–response analyses to study the impact of other signalling cascades on the expression of the identified TFs, and steady-state analysis revealing combinations and conditions that can switch on or off the identified TFs, mimicking the effects of the treatment [23].

## 2. Materials and Methods

### 2.1. Data Description and Pre-Processing

We used a transcriptomic dataset of white blood cells from RA patients and healthy donors (GSE117769) sequenced at CSL Limited/bio21 Institute (30 Flemington Rd, Parkville, Australia) using the Illumina HiSeq 2500 platform (Illumina, Inc.). The dataset describes 120 subjects in total (51 RA and 50 controls, and 19 patients with either ankylosing spondylitis or psoriatic arthritis). From this dataset, we extracted 96 samples (46 RA and 50 controls). For the 46 RA samples, 43 were of Caucasian ancestry and 3 were of unknown ancestry. Moreover, two samples consisted of duplicates of the exact origin (female, control, unknown ancestry), and their expression matrix average was used for the analysis. We conducted a preliminary analysis on the expression matrix data with DESeq2 version 1.32.0 [24], using normalisation and variance stabilising transformation on the matrix expression. Principal Component Analysis (PCA) revealed five outliers (4 RA and one control) that were removed from the dataset (Figure S1). The final dataset used for further analysis comprised 90 samples (42 RA and 48 controls). Finally, we performed a normalisation on the raw expression matrix after removing low read counts (>10).

### 2.2. Inference of the Co-Regulatory Network (CoRegNet)

We used CoRegNet [17] R package version 1.26.0 to infer the co-regulatory network with the normalised gene expression matrix of the pre-processing step. The CoRegNet package implements the H-LICORN algorithm, allowing identifying cooperative gene regulators [25]. We enriched the inferred network with protein–protein and regulation evidence and further refined it with an unsupervised method using the unweighted mean. The co-regulatory network inferred with CoRegNet is composed of the significant edges between TF with a False Discovery Rate (FDR) [26] of 5%.

### 2.3. RA Map Upstream Protein Extraction

The RA map is a state-of-the-art interactive knowledge base for the RA disease [18]. The RA map is organised in the form of a cell representing the flow of information from the extracellular space to the plasma membrane and then to the cytoplasm, the nucleus, and the secreted compartment or cellular phenotypes. For our analysis, as we were focused on upstream regulators of identified TFs, we mainly used the RA map’s signalling part. More specifically, the list of TFs was uploaded as an overlay to the RA map, and the matching TFs were identified. The matching TFs were subsequently used as seeding nodes for the upstream plugin [27], setting the mode of extraction as upstream and selecting non-blocking modifiers. Finally, the obtained file was extracted as an XML CellDesigner file.

With this method, we extracted upstream signalling cascades and seven translation reactions for which the mRNA was directly linked with the protein in the cytoplasm or the membrane. The RA map, and consequently the extracted network, is written in the Process Description Systems Biology Graphical Notation scheme [28]. To obtain a more simplified representation of the network, the CellDesigner XML file of the previous step was used as an input to the tool CaSQ [29] to create an Activity Flow (AF)-like executable network. CaSQ provides SBML-qual files for performing *in silico* simulations, but in our case, we used only the SIF file that contains information about the source, interaction type, and target of the Boolean network.

The obtained SIF file was further modified to address the issue of complexes. First, we recreated the reactants for every complex represented as a single node in the AF network. This way, we would not miss interactions and overlaps between nodes existing inside complexes. On the other hand, regarding entities represented multiple times (as genes, proteins, or mRNAs), we kept only one entity for simplification purposes and merged the corresponding interactions.

### 2.4. Global RA Network Inference

We used the R package igraph [30] to convert the CoRegNet object (co-regulatory network) and the RA map SIF file into separate graphs. Then, we merged both networks using igraph and imported the network into Cytoscape using the RCy3 R/Bioconductor package [31], forming the global RA-specific network.

### 2.5. Differential Expression Analysis (DEA) Using Independent Datasets

We conducted multiple DEA using two different datasets. One dataset contained normalised counts from RNA sequencing data of CD4+ T cells, including different responses of RA patients to anti-TNF treatment (GSE138747). The dataset comprises two cohorts of RA patients treated with adalimumab (37 patients) and etanercept (41 patients), which were analysed independently. The second dataset comprises raw counts from RNA sequencing data of whole blood cells of biologic naive RA patients from baseline and after three months of treatment with infliximab or adalimumab (GSE129705). This dataset contains two different cohorts of 40 and 36 RA patients, which were also analysed independently. Thus, using DESeq2, we conducted two DEA on comparing responders and non-responders’ gene expression levels for both drugs (adalimumab and etanercept) and two DEA on the comparison of baseline and after three months of anti-TNF treatment gene expression level. We considered as differentially expressed genes (DEG) the ones with a corrected *p*-value (FDR) < 0.1 for all performed analyses. The DEG lists were used as an overlay for the global RA-specific network.

### 2.6. List of Variants

DisGeNET [32] contains the most extensive publicly available collection of genes and variants associated with human diseases. From this database, we extracted 2387 variants associated with RA. Then, we filtered out variants with a variant disease association (VDA) and evidence index (EI) score lower than 0.7. The VDA score is computed using the number of curated and non-curated publications supporting the variant disease association, while the EI score is computed using contradictory results in publications supporting the variant. A 0.7 threshold gives us at least one curated publication supporting the variant and the disease association resulting in 1635 variants. Within these 1635 variants, we identified 731 associated genes that were subsequently used as an overlay for the global RA-specific network.

### 2.7. Subnetwork Extraction

The subnetwork, based on the global network for RA, is focused on Tumor Necrosis Factor (TNF), Interleukin 6 (IL6), and Transforming Growth Factor Beta 1 (TGFB1), which are three molecules highly implicated in RA (see Section 4). These three proteins were extracted with their downstream cascades up to the first TF to reduce complexity and focus on the upstream regulators. From the global network for RA displayed in Cytoscape, we selected TNF, IL6, and TGFB1 simultaneously and using the Biological Network Manager (BiNoM) plugin, we selected in a stepwise manner the downstream neighbours of TGFB1, IL6, and TNF up to the first affected TF(s).

### 2.8. Shiny App

The co-regulatory network inferred with CoRegNet, the RA map Activity Flow extracted network, and the merged global RA network, along with their overlays, were integrated into a web-based Shiny application using R [33]. The web application uses a Cytoscape viewer based on the R package cyjShiny [34] and is freely available (https://quentin-miagoux.shinyapps.io/global_ra_network (accessed on 1 July 2021)).

### 2.9. Inference of a Boolean Network for In Silico Simulations

The subnetwork obtained in Section 2.7 was imported in Cytoscape and exported in SBML format using the BiNoM plugin and its function “export to SBML”. The file was subsequently imported in CellDesigner to adjust the layout and remove co-regulatory interactions between TFs to focus only on upstream regulators. Finally, the CellDesigner SBML file was used to infer a Boolean model in an SMBL-Qual format using CaSQ (CellDesigner as SBML-Qual) v0.9.11.

Then, the SMBL-Qual file was imported into Cell Collective to perform real-time simulation experiments. Using the **“***Simulation***”** tab on Cell Collective, we mimicked the downregulation of the components in the datasets used (before/after treatment, responders/non-responders, mutation carriers) under different initial conditions for each input (TNF, IL6, and TGFB1). Furthermore, we performed sensitivity and dose–response analyses using five different initial conditions described in Table 3.

The same SBML-Qual file was also used for analysis with the software GINsim [35] after a post-processing modification step for node name recognition. We used the nightly build version 3.0.0b-SNAPSHOT, and the functions *Reduce model* for the reduced version, *Compute stable states* to obtain the stable states of the model and *Run simulation* with configurations of perturbations for the *in silico* knock out (KO) experiments.

## 3. Results

### 3.1. Inference of the Co-Regulatory Network

**Figure 2:**
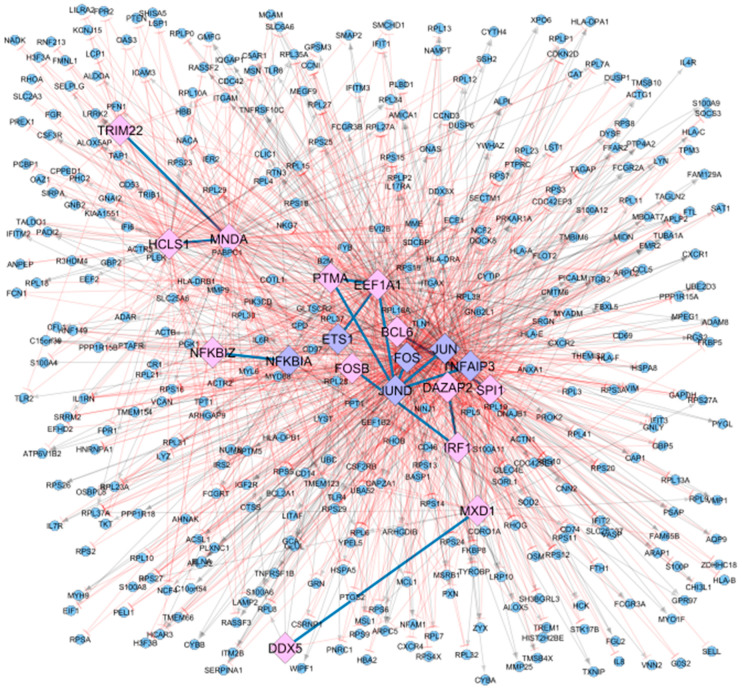
Co-regulatory network inferred using the tool CoRegNet and the matrix of normalised counts from the transcriptomic dataset (GSE117769). The dataset used included data from 90 samples (48 Controls and 42 RA patients). Matching TFs from CoRegNet with the RA map are depicted using a diamond shape and coloured in purple (6), while non-matching TFs are depicted in a diamond shape and coloured in pink (13). CoRegNet target genes are coloured in blue (373) and have round shapes. Inhibitions are represented with blunt red arrows, and activations are represented with grey arrows.

We selected a transcriptomic dataset from the GEO database (GSE117769) to infer the co-regulatory network, including 120 samples (51 RA and 50 control, and 19 patients with either ankylosing spondylitis or psoriatic arthritis). After a series of pre-processing checks, including the sample origins, duplicates, and quality of the data using a PCA on the matrix expression with normalisation and variance stabilising transformation (shown in Figure S1), we kept for further analysis a total of 90 samples (48 Controls and 42 RA patients). Then, of the remaining samples, we obtained normalised counts using DESeq2, on which we finally applied CoRegNet to infer the co-regulatory network, which is presented in Figure 2.

This network includes a total of 19 TFs, 14 co-regulatory interactions, and a total of 373 regulated target genes. Table 1 summarises the top five TFs with the highest number of regulatory and co-regulatory interactions. The literature search for the nineteen TFs identified from CoRegNet as the master regulators in the dataset showed their potential implication to RA. Supplementary Table S1 summarises key roles of the TFs and the corresponding literature reference.

### 3.2. RA Map Upstream Regulators of the TFs Identified from CoRegNet

**Figure 3:**
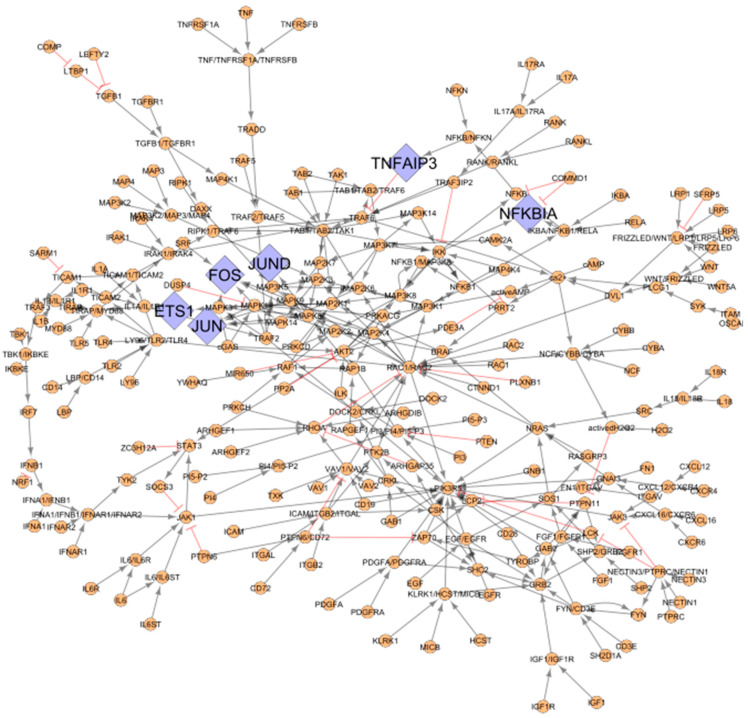
The upstream regulators of the matching TFs between the RA map and the CoRegNet co-regulatory network. Matching TFs from the inferred network with CoRegNet are coloured in purple (diamond shape) (6), and upstream regulators (round shape) are coloured in orange (238). Inhibitions are represented with blunt red arrows and activations with grey arrows.

Six out of the 19 TFs, namely ETS Proto-Oncogene 1, Transcription Factor (ETS1), Fos Proto-Oncogene, AP-1 Transcription Factor Subunit (FOS), Jun proto-oncogene, AP-1 transcription factor subunit (JUN), JunD Proto-Oncogene, AP-1 Transcription Factor Subunit (JUND), NFKB Inhibitor Alpha (NFKBIA), and TNF Alpha Induced Protein 3 (TNFAIP3) from the co-regulatory network are present in the RA map. Therefore, they were used as seeds to extract their upstream regulators. The extracted network comprising the RA map upstream regulators of the matching TFs includes 244 nodes, as shown in Figure 3.

### 3.3. Coupling Gene Co-Regulation with Signalling Cascades to Obtain a Global, Integrative RA Network

**Figure 4:**
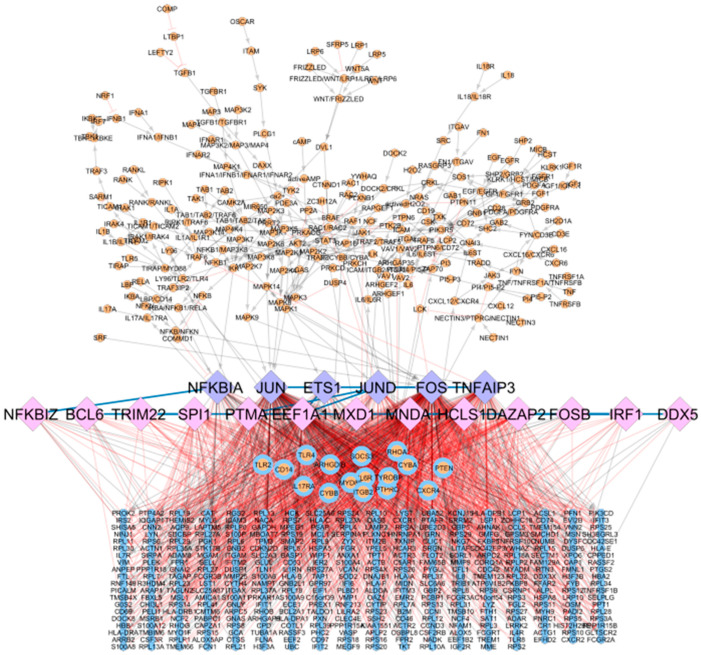
Integrative global network for rheumatoid arthritis. RA map upstream regulators are bound to TFs identified in the CoRegNet co-regulatory network. Matching TFs are coloured in purple (6), matching genes/proteins are coloured in blue and orange (16), upstream regulators from the RA map are coloured in orange (222), CoRegNet TFs are coloured in pink (13), and CoRegNet target genes are coloured in blue (357). Inhibitions are represented with blunt red arrows, and activations are represented with grey arrows. Transcription factors are depicted in diamond shapes, while upstream regulators and target genes are depicted using round shapes.

The global, integrative RA network results from merging the RA map signalling cascades and the CoRegNet object, using as an interface the matching TFs. It comprises 614 nodes and 1736 interactions (848 inhibitions, 874 activations, and 14 co-regulatory interactions shared among TFs), including genes, proteins, complexes, and simple molecules shown in Figure 4. In this network, six TFs were shared between the CoRegNet network and the RA map (seeding TFs). In addition, 16 target genes identified with CoRegNet overlapped with the RA map upstream regulators.

### 3.4. Two Use Cases: Identification of Key TFs Using DEG from RA Patients Undergoing Anti-TNF Treatment

Two datasets of RNAseq expression data coming from RA patients undergoing anti-TNF treatment were analysed to obtain DEG. The first dataset focuses on responders and non-responders to RA treatment, including 37 and 41 RA patients treated with adalimumab and etanercept, respectively. The second one involves untreated and treated (infliximab or adalimumab) RA patients, including two cohorts of 40 and 36 RA patients. DEGs from these analyses were mapped to the global network for RA (presented in Figures S2 and S3, respectively).

DEGs from responders/non-responders RA patients data mapping shows a total of 15 matching nodes, including 4 etanercept DEGs and 11 adalimumab DEGs. In addition, four matching nodes are CoRegNet and RA map TFs (NFKBIA, JUN, FOS, and TNFAIP3) and 1 CoRegNet TF only (FOSB), in the global network for RA DEGs from untreated and treated RA patients mapping show a total of 101 matching nodes, including 2 CoRegNet and RA map TFs (NFKBIA and FOS) and 4 CoRegNet TF only (BCL6 Transcription Repressor (BCL6), MAX Dimerisation Protein 1 (MXD1), Myeloid Cell Nuclear Differentiation Antigen (MNDA), and DAZ-Associated Protein 2 (DAZAP2)).

Finally, cross-analysis revealed that a total of 9 over 19 TFs included in the global network for RA overlapped with a DEG from at least one analysis (presented in Table 2). Among these 9 TFs, two of them, NFKBIA and FOS, overlapped with a DEG in both analyses.

### 3.5. Logic-Based Dynamical Analysis of the Subnetwork

While the analyses highlight TFs differentially expressed (downregulated) after treatment or response to treatment with anti-TNF drugs, it is evident from the global network that the identified TFs can be regulated by a variety of other upstream cascades, besides those implicated in the TNF signalling.

To study further the interconnections with other pathways, we focused on IL6 and TGF-beta signalling (mentioned as TGFB1 in the network). IL6 is the target of tocilizumab (TCZ), which is an IL6 inhibitor frequently used in the treatment of RA. The inhibitor was developed in 2008, and its therapeutic efficacy is quite similar to those of TNF inhibitors [36]. TGF-beta signalling is activated in RA synovium; however, TGF-beta blockade did not seem to affect experimental arthritis [37]. To study further the impact of these cascades on the expression of the identified TFs, we constructed a subnetwork by selecting the molecules TGFB1, IL6, and TNF in the global network along with their downstream neighbours until reaching an identified TF.

**Figure 5:**
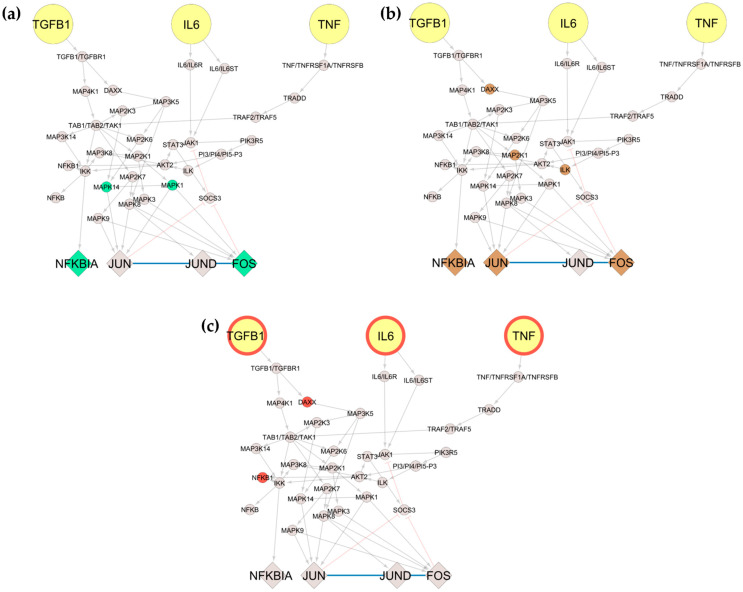
Subnetwork extraction of target molecules (TGFB1, IL6, and TNF) from the global network for RA. We focused on TGFB1, IL6, and TNF (coloured in yellow) and their downstream neighbours (depicted in round shapes) until reaching the first transcription factor (depicted in diamond shapes). Then, we projected on the network (**a**) DEG from responders/non-responders RA patients to anti-TNF treatment, as shown in green (4); (**b**) DEG after/before anti-TNF treatment of RA patients is shown in brown (6); and (**c**) DisGeNET variants are shown in red (5).

The subnetwork contains 38 nodes, including 4 TFs and is highly enriched in MAPKs, as seen in Figure 5. By projecting the DEGs and known genomic variants associated with the disease, we can see that intermediate nodes and TFs are downregulated by the anti-TNF treatment, while the inputs (IL6, TNF, and TGFB1) along with a few intermediate nodes are characterised as mutation carriers.

In the next step, we wanted to evaluate the impact of single and combined perturbations of the network inputs on the expression of the TFs. We used the possibility of adding Boolean rules to the network with the tool CaSQ [29]. Boolean models have been long used to describe biological mechanisms in health and disease [38], and they are an optimal approach for modelling signalling and gene regulation when kinetic parameters are scarce. Boolean models use binary values and logical operators (AND, OR, and NOT) to describe the regulation of all molecules in the system [21]. The CaSQ tool receives an SBML CellDesigner file [39] and produces a Boolean network with preliminary logical rules.

The Boolean model produced from our subnetwork has 38 nodes (three inputs, six outputs and 29 intermediate nodes) and 59 interactions. The SBML qual file was imported to Cell Collective [40] to perform real-time simulations and sensitivity analysis and GINsim [35] to calculate stable states and perform *in silico* KO simulations. For the *in silico* KO simulations, a reduced version of 23 nodes was used.

### 3.6. Real-Time Simulations Using the Cell Collective Platform

First, we wanted to see the impact of the molecules, either affected by the treatment or identified as mutation carriers, on the model outputs. Before and after anti-TNF treatment, the analysis showed that Mitogen-Activated Protein Kinases such as MAPK14 and MAPK1 were downregulated. Accordingly, for the responders and non-responders’ analysis, Mitogen-Activated Protein Kinase Kinase 1 (MAP2K1), Integrin Linked Kinase (ILK), and Death Domain-Associated Protein (DAXX) were also identified as downregulated. Lastly, DAXX and Nuclear Factor Kappa B Subunit 1 (NFKB1) were identified as mutation carriers. To mimic the effects of the downregulation of these molecules on the model outputs, we performed *in silico* simulations setting their activation level to zero.

**Figure 6:**
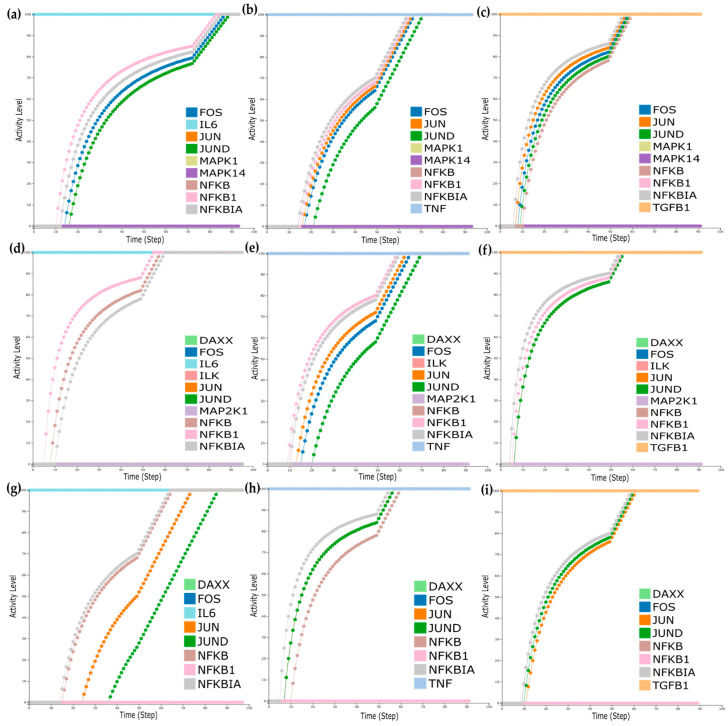
Real-time simulations with Cell Collective for the dataset of before and after anti-TNF treatment of RA patients, where MAPK14 and MAPK1 were found to be downregulated (**a**–**c**); (**a**) simulations with IL6 activity set to 100%, (**b**) simulations with TNF activity set to 100%, (**c**) simulations with TGFB1 activity set to 100%. Real-time simulations for the dataset of responders/non-responders where MAP2K1, ILK, and DAXX were found downregulated (**d**–**f**); (**d**) simulations with IL6 activity set to 100%, (**e**) simulations with TNF activity set to 100%, (**f**) simulations with TGFB1 activity set to 100%. Real-time simulations for the dataset of the mutation carrier. (**g**–**i**); (**g**) simulations with IL6 activity set to 100%, (**h**) simulations with TNF activity set to 100%, (**i**) simulations with TGFB1 activity set to 100%.

For the dataset of before and after anti-TNF treatment of RA patients, MAPK14 and MAPK1 activity levels were set to zero, and simulations turning the inputs sequentially active revealed that when setting either TNF, TGFB1, or IL6 on, all TFs are expressed (Figure 6a–c). Furthermore, when mimicking the downregulation of MAP2K1, ILK, and DAXX for the dataset of responders/non-responders to anti-TNF treatment, we observed that when setting TNF and TGFB1 on, all TFs are expressed (Figure 6d,e). However, when IL6 is set on, only the TF NFKBIA is expressed (Figure 6f). Finally, when mimicking the downregulation of DAXX and NFKB1 for the mutation carrier from DisGeNET, we observe that when setting either TNF, TGFB1, or IL6 on, all TFs are expressed (Figure 6g–i).

### 3.7. Dose–Response and Sensitivity Analysis

For the dose–response, we studied five different initial conditions shown in Table 3 that mimic different scenarios’ effects in combination with TNF activity status. The first condition corresponds to TNF blockade and simultaneous impairment of IL6 and TGFB1 signalling; the second corresponds to having the IL6 cascade active, the third to having the TGFB1 active, the fourth to having both IL6 and TGFB1 active, and lastly, the fifth condition mimics what happens to the system when only the TNF input is active.

**Figure 7:**
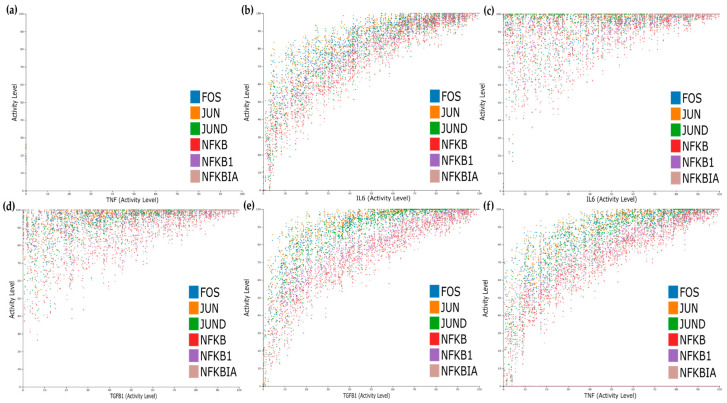
Dose–response analysis. (**a**) All inputs inactive. (**b**) IL6 active. (**c**) IL6 and TGFB1 active (TGFB1 view). (**d**) IL6 and TGFB1 active (IL6 view). (**e**) TGFB1 active. (**f**) TNF active.

We performed dose–response analysis for all conditions and observed that the expression of the TFs is dose-dependent for TNF, TGFB1, and IL6 (Figure 7b,e,f), while the simultaneous activation of IL6 and TGFB1 cascades has a synergistic effect causing an increase on the activation levels of the TFs even for lower doses of IL6 and TGFB1 (Figure 7c,d).

Next, we wanted to see how the downregulation of the TFs observed after the anti-*TNF* treatment could be counterbalanced by the other pathways, given that in non-responders, the expression of these TFs was kept intact, despite the administered treatment. Therefore, we performed an environment sensitivity analysis to identify which of the two model inputs (IL6 and TGFB1) has the most significant impact on the up-regulation of the TFs included in the model when TNF activity is blocked. The results showed that the TFs could be upregulated in the absence of TNF activity for a combination of activity ranges of the other two inputs (Supplementary Materials: Figure S4).

### 3.8. Wild-Type Stable-State Analysis and KO Simulations

**Figure 8:**
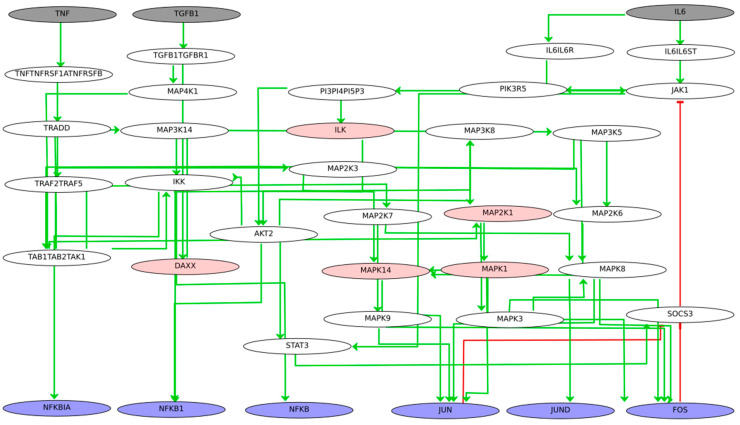
The Boolean network comprising the three signalling cascades for TNF, TGFB1, and IL6. Boolean rules were inferred using the tool CaSQ (see Section 2 for a step-by-step model inference). Inputs are depicted in grey, TFs of interest are depicted in purple, and intermediate nodes affected by the drug treatment or identified as mutation carriers are depicted in pink. Green arrows denote activation, and blunt red arrows denote inhibition.

We performed stable-state analysis for the model using the software GINsim (Figure 8). The analysis for the wild type (no perturbations) revealed five steady states (fixed points) and no complex attractor. The configurations of these five stable states as far as the molecules of interest are concerned (grey, blue, and pink nodes of Figure 8) are shown in Table 4.

The analysis shows that for the TFs identified as master regulators, the activation of IL6 or IL6 and TGFB1 can positively regulate their expression, even in the presence of the anti-TNF treatment (TNF = 0). Blocking the TNF cascade can completely shut down their expression only if combined with the blocking of IL6 and TGFB1 cascades. Regarding DAXX, it is actively expressed only when TGFB1 is activated, and ILK is dependent on the activation of IL6. The MAPK molecules are dependent on the activation of IL6 and TGFB1 and do not seem to be impacted by TNF blocking.

Next, we created a reduced version of the Boolean modelling using the reduction function of the software GINsim to perform *in silico* experiments with combined perturbations. The reduced Boolean model comprised 23 nodes, and for the analysis, we created virtual KOs for (a) MAPK14 and MAPK1, (b) DAXX, ILK, and MAP2K1, and (c) DAXX and NFKB1 to mimic the effects of the anti-TNF treatment and the mutation carriers, which were identified previously. For the simulations, we set the initial conditions for the TNF to zero and let the other inputs free, while setting the initial condition for all intermediate nodes to zero.

The results of the *in silico* experiments for the molecules of interest and the three conditions are shown in Table 5, Table 6 and Table 7. For each set of conditions, the system was able to reach three steady states. For the first set of conditions, we observe that besides DAXX that is strictly TGFB1 dependent (Table 5, ss2 and ss3), all TFs can get activated with the presence of IL6 or IL6 and TGFB1, despite the TNF blockade and the downregulation of MAPK14 and MAPK1.

For the second set of conditions, we observe that when TNF is blocked and DAXX, ILK, and MAP2K1 are downregulated, the IL6 signal alone is not enough to activate the TFs JUN, FOS, and JUND and the kinases MAPK14 and MAPK1 (Table 6, ss2). However, when both IL6 and TGFB1 signals are on, all TFs are activated, and the activity level of kinase MAPK14 is restored (Table 6, ss3).

Lastly, we simulated the effects of the mutation carriers, as identified by DisGeNet, and the TNF blockade on the activity of the TFs and the kinases in our network. In Table 7, we observe that despite TNF blockade and DAXX and NFKB1 downregulation, all identified TFs and kinases are activated in the presence of IL6 or for IL6 and TGFB1 combined activity.

## 4. Discussion

In the present work, we combine gene co-regulation with mechanistic signalling cascades to provide information about upstream regulation. Furthermore, we use the integrative RA network to analyse transcriptomic data regarding anti-TNF treatment and map information about known disease-associated mutation carriers. Lastly, we use the tool CaSQ to add Boolean dynamics to a subnetwork of interest to mimic the effects of the anti-TNF treatment and estimate the impact of IL6 and TGFB1 and the downregulated genes on the activation profile of the identified TFs.

The nineteen TFs identified as master regulators have been implicated in RA and autoimmunity, as the literature evidence supports. Six out of the nineteen TFs were also present in the RA map, which is a state-of-the-art mechanistic network for the disease built using manual curation. These six TFs, namely JUN, JUND, FOS, NFKBIA, ETS1, and TNFAIP3, were used as a functional overlap between the co-regulation and the signalling events, enabling us to obtain a network comprising upstream cascades, active TFs, and target genes.

We used the integrative network as a template to analyse two independent datasets regarding anti-TNF treatment. First, we observed the downregulation of some of the TFs previously identified as master regulators. Second, to study the impact of the treatment in parallel with the activity of other signalling cascades, we extracted subgraphs from the integrative network. Finally, we selected the cascades of TNF, IL6, and TGFB1, up to the first affected TF to reduce complexity and focus on the upstream regulators.

We selected IL6, as it is one of the targets of the biologic treatment in RA [36,41,42,43] and TGF-beta because it is an immunomodulatory cytokine highly expressed in RA patients, with a role that is yet to be determined [44,45,46]. We adjusted the map-to-model framework described in Aghamiri et al. [29] to obtain an executable Boolean subnetwork to perform *in silico* analysis. As demonstrated from the real-time simulations and the dose–response analysis, both IL6 and TGFB1 cascades could affect the expression of the TFs, and as seen from the component sensitivity analysis, IL6 and TGFB1 could even counterbalance the downregulation of the studied TFs caused by the TNF blockade.

The steady-state analysis confirmed the real-time simulation results showing that for the TFs identified as master regulators, the activation of IL6 or IL6 and TGFB1 cascades can positively regulate their expression. Blocking the TNF cascade can completely shut down the expression of these TFs only if combined with the blocking of IL6 and TGFB1 cascades. Towards this direction, dual-targeted therapies have been proposed, either with the development of dual-target agents, blocking IL6 and TNF simultaneously, for example [47], or by administering two biologics at the same time. However, the administration of combined biologics has been linked to increased adverse effects, and it is currently under study to evaluate better dosage schemes [48,49].

Simulations with combined KOs mimicking the effect of anti-TNF treatment in combination with the downregulation of genes observed in the analysed datasets confirmed the dependency of the TFs activation state on the presence of inputs and further highlighted specific conditions. For example, when TNF is blocked, and DAXX, ILK and MAP2K1 are downregulated, the IL6 signal alone is not enough to activate the TFs JUN, FOS, JUND, and the kinases MAPK14 and MAPK1 (Table 6, ss2). However, when both IL6 and TGFB1 signals are on, all TFs are activated, and the activity level of kinase MAPK14 is restored (Table 6, ss3). MAPK14 (p38a kinase) and MAPK1 (ERK2) are two proteins known to play a pivotal role in RA and are activated by a variety of signals, including cytokines such as TNF and IL6 but also TGF-beta [50]. While p38 had been proposed as a potential target to reduce the destruction of bone and cartilage, p38 inhibitors have given disappointing results regarding therapeutic efficacy [51,52].

As seen in Table 5, the suppression of MAPK14 (p38a) does not inhibit the activation of the identified as master regulators TFs, even in the presence of anti-TNF treatment, as other inputs, such as IL6 or TGF-beta, can counterbalance the effects. Regarding ERK inhibitors, limited data are available, which may be due to a lack of efficient pharmacological inhibitors [53]. In older studies, FR180204, an ERK inhibitor, had demonstrated effectiveness against mouse collagen-induced arthritis [54], but there was no significant follow-up. In our model, ERK2 inhibition (MAPK1) does not seem to significantly impact the activation of its downstream target TFs, JUN, and FOS, as other regulators can also activate them.

## 5. Conclusions

While resistance to TNF therapy is a common event in the treatment of RA, the reasons behind its mechanisms are still unclear [55]. In addition, currently, there is no way of predicting which patient will respond or not to targeted therapy [56].

While the heterogeneity in RA is evident as manifested by the different patient profiles, the affected molecular pathways involved in the disease and autoimmunity are well known and studied. Therefore, a way to address the heterogeneity is to create backbone models comprising all affected pathways derived from the literature and big data to obtain global blueprints of the perturbed cascades. Then, patient-specific data can be used to contextualise each model by highlighting affected biomolecules (genes, proteins, metabolites, etc., depending on the type of data). In this way, patient-specific models could be created based on integrated, personalised data, such as clinical information, comorbidities, and genetic factors (mutations in specific genes). In addition, single-cell datasets could also provide insights into the disease heterogeneity at the cellular level.

Executable, integrated networks can accelerate the building of personalised models, as mapping dysregulated genes could reveal potentially impacted pathways shedding light on therapy response. Dynamic analysis and *in silico* simulations can also inform about the outcome of combined perturbations, predicting the emergent behaviour of the system. Integrating multi-omics data is a key step in understanding pathogenetic mechanisms of multifactorial diseases, where one level of information does not suffice to explain the complex phenotypic traits. Integrative networks allow for patient-level analysis by using patient-specific data and analysing the effects of patient-specific mutations and DEGs, combined with treatment effects. Such approaches could inform on the possibilities of success of a given therapy. For example, one could test the effects of mono or combined therapy, such as methotrexate (MTX) and anti-TNF, to better evaluate possible responses. Larger patient cohorts and more efficient computational techniques that would allow simulations on a larger scale could enhance the robustness and the predictive power of such models, helping to understand the response or non-response to a given therapy at a patient level.

## Figures and Tables

**Table 1:** Top 5 of the CoRegNet identified transcription factors (TFs) with the highest number of regulatory interactions (TF–target gene) and co-regulatory interactions (TF–TF). The analysis was performed using data from 90 samples, 48 Controls, and 42 RA patients.

**Top 5 TFs**	**No. of Regulatory Interactions**
FOS	288
JUN	211
EEF1A1	155
MNDA	136
TNFAIP3	125
**Top 5 TFs**	**No. of Co-Regulatory Interaction(s)**
JUND	5 (EEF1A1, FOS, JUN, PTMA, TNFAIP3)
EEF1A1	3 (ETS1, JUND, PTMA)
IRF1	2 (DAZAP2, FOSB)
MNDA	2 (HCLS1, TRIM22)
PTMA	2 (EEF1A1, JUND)

**Table 2:** Transcription factors from the global network for RA with at least one differentially expressed gene overlapping from the analysis of (a) responders/non-responders RA patients to anti-TNF treatment (37 and 41 RA patients treated with adalimumab and etanercept, respectively) and (b) after/before anti-TNF treatment of RA patients (two different cohorts of 40 and 36 RA patients from baseline and after three months treatment with Infliximab or Adalimumab). ↓ denotes downregulation.

Source	TF	Responders/Non-Responders	After Treatment/Before Treatment
CoRegNet and RA map	NFKBIA	↓	↓
	JUN		↓
	FOS	↓	↓
CoRegNet	TNFAIP3		↓
	BCL6	↓	
	MXD1	↓	
	MNDA	↓	
	DAZAP2	↓	
	FOSB		↓
	NFKBIA	↓	↓
	JUN		↓

**Table 3:** Initial conditions for dose–response analysis.

Initial Conditions	Input State
1	All inputs inactive
2	IL6 active
3	TGFB1 active
4	IL6 + TGFB1 active
5	TNF active

**Table 4:** Stable states of the Boolean network (wild type).

Steady States	TNF	IL6	TGFB1	JUN	FOS	JUND	NFKBIA	DAXX	ILK	NFKB1	MAP2K1	MAPK1	MAPK14
ss1	0	0	0	0	0	0	0	0	0	0	0	0	0
ss2	0	1	0	1	1	1	1	0	1	1	1	1	1
ss3	0	1	1	1	1	1	1	1	1	1	1	1	1
ss4	1	1	0	1	1	1	1	0	1	1	1	1	1
ss5	1	1	1	1	1	1	1	1	1	1	1	1	1

**Table 5:** Stable states of the Boolean network (MAPK1, MAPK14 KO, input TNF = 0).

Steady States	TNF	IL6	TGFB1	JUN	FOS	JUND	NFKBIA	DAXX	ILK	NFKB1	MAP2K1	MAPK1	MAPK14
ss1	0	0	0	0	0	0	0	0	0	0	0	0	0
ss2	0	1	0	1	1	1	1	0	1	1	1	0	0
ss3	0	1	1	1	1	1	1	1	1	1	1	0	0

**Table 6:** Stable states of the Boolean network (DAXX, ILK and MAP2K1 KO, input TNF = 0).

Steady States	TNF	IL6	TGFB1	JUN	FOS	JUND	NFKBIA	DAXX	ILK	NFKB1	MAP2K1	MAPK1	MAPK14
ss1	0	0	0	0	0	0	0	0	0	0	0	0	0
ss2	0	1	0	0	0	0	1	0	0	1	0	0	0
ss3	0	1	1	1	1	1	1	0	0	1	0	0	1

**Table 7:** Stable states of the Boolean network (DAXX and NFKB1 KO, input *TNF* = 0).

Steady States	TNF	IL6	TGFB1	JUN	FOS	JUND	NFKBIA	DAXX	ILK	NFKB1	MAP2K1	MAPK1	MAPK14
ss1	0	0	0	0	0	0	0	0	0	0	0	0	0
ss2	0	1	0	1	1	1	1	0	1	0	1	1	1
ss3	0	1	1	1	1	1	1	0	1	0	1	1	1

## Data Availability

Datasets (GSE117769, GSE129705, GSE138747, and DisGeNET variants) used for the analysis are publicly available. All data and code used to generate results, including networks inference, differential expression analysis, network visualisation, and Boolean model simulation, are available on a GitLab repository at https://gitlab.com/genhotel/inference-of-a-global-integrative-network-for-rheumatoid-arthritis (accessed on 1 July 2021). The Shiny app is freely available at https://quentin-miagoux.shinyapps.io/global_ra_network (accessed on 1 July 2021).

## Supplementary Materials

The following are available online at https://www.mdpi.com/article/10.3390/jpm11080785/s1, Figure S1: Principal component analysis (PCA) in samples of human blood cells from RA patients and healthy controls. The PCA shows 95 samples from the GSE117769 dataset (46 RA samples and 49 controls). In addition, a variance stabilising transformation was carried on the matrix expression data. Figure S2: Global RA network and DEG from responders/non-responders to anti-TNF treatment (37 and 41 RA patients treated with adalimumab and etanercept, respectively). Overlapping DEG from adalimumab treatment and etanercept treatment data are shown in brown (11) and pink (4), respectively, while non-overlapping genes/proteins are shown in grey (599). Transcription factors are depicted in diamond shapes, while upstream regulators and target genes are depicted using round shapes. Figure S3: Global RA network and DEG from untreated and treated RA patients with anti-TNF treatment (two different cohorts of 40 and 36 RA patients from baseline and after three months treatment with infliximab or adalimumab). Overlapping DEG are shown in green (101), while non-overlapping genes/proteins are shown in grey (513). Transcription factors are depicted in diamond shapes, while upstream regulators and target genes are depicted using round shapes. Figure S4: Environment sensitivity analysis. Transcription factors (TFs) could be upregulated in the absence of TNF activity for a combination of activity ranges of the other two inputs (IL6 and TGFB1). The upper part of each subfigure shows the impact of the external components on the activity state of the selected TFs, and the lower part of the image shows the range of activity percentage of the external components to achieve the optimisation of the activity state of the selected TFs. Sensitivity analysis for (**a**) JUND; (**b**) JUN; (**c**) FOS; (**d**) NFKB1; (**e**) NFKBIA; boxplots of IL6 in blue, TGFB1 in orange, and TNF as black line as it is set to off. Table S1: Transcription factors identified from CoRegNet and their involvement in RA based on literature evidence.
