# Supplementary material for: Inference of an Integrative, Executable Network for Rheumatoid Arthritis Combining Data-Driven Machine Learning Approaches and a State-of-the-Art Mechanistic Disease Map
Source: J Pers Med. 2021 Aug 12;11(8):785. doi: 10.3390/jpm11080785 (PMC8400381; doi:10.3390/jpm11080785)
Supplement: Supplementary file 1 [file jpm-11-00785-s001.zip › jpm-1315161-supplementary.pdf]

Inference of an integrative, executable network for Rheumatoid Arthritis combining data-driven machine learning approaches and a state-of-the-art mechanistic disease map

Quentin Miagoux[1], Vidisha Singh[1], Dereck de Mezquita[1], Valerie Chaudru[1], Mohamed Elati[2], Elisabeth Petit-Teixeira[1], Anna Niarakis[1,3]

1 Université Paris-Saclay, Univ Evry, Laboratoire Européen de Recherche pour la Polyarthrite rhumatoïde - Genhotel, 91057, Evry, France
2 CANTHER, University of Lille, CNRS UMR 1277, Inserm U9020, 59045 Lille, France
3 Lifeware Group, Inria Saclay-île de France, Palaiseau 91120, France

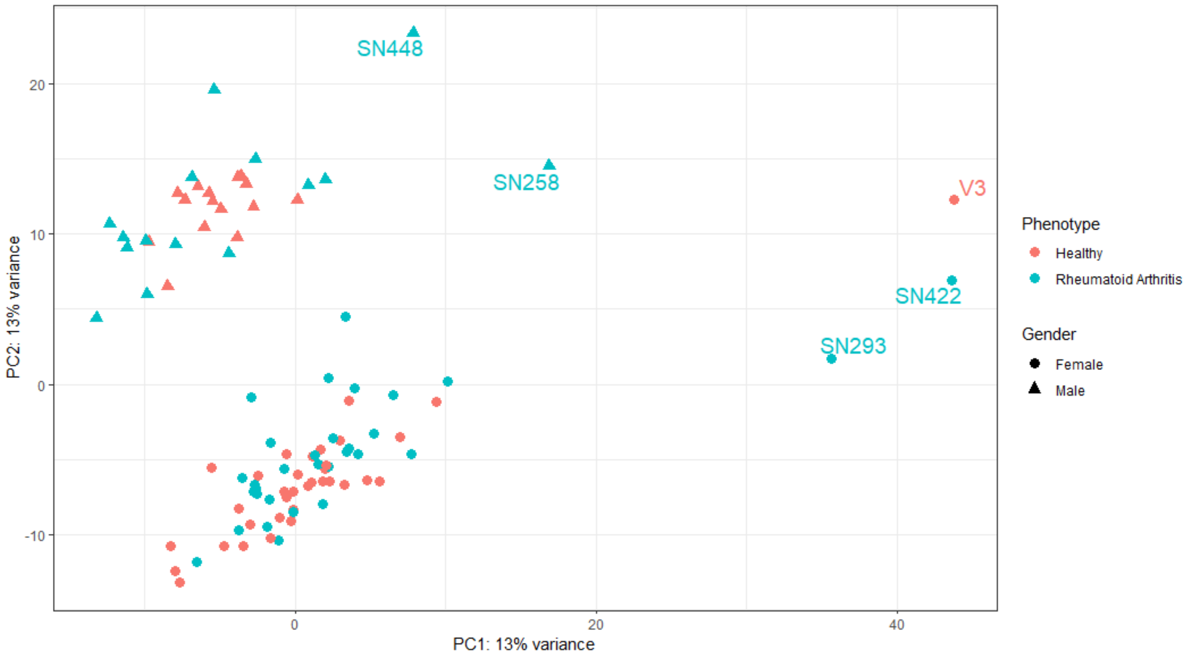

Figure S1. Principal component analysis (PCA) in samples of human blood cells from RA patients and healthy controls. The PCA shows 95 samples from the GSE117769 dataset (46 RA samples and 49 controls). In addition, a variance stabilising transformation was carried on the matrix expression data.

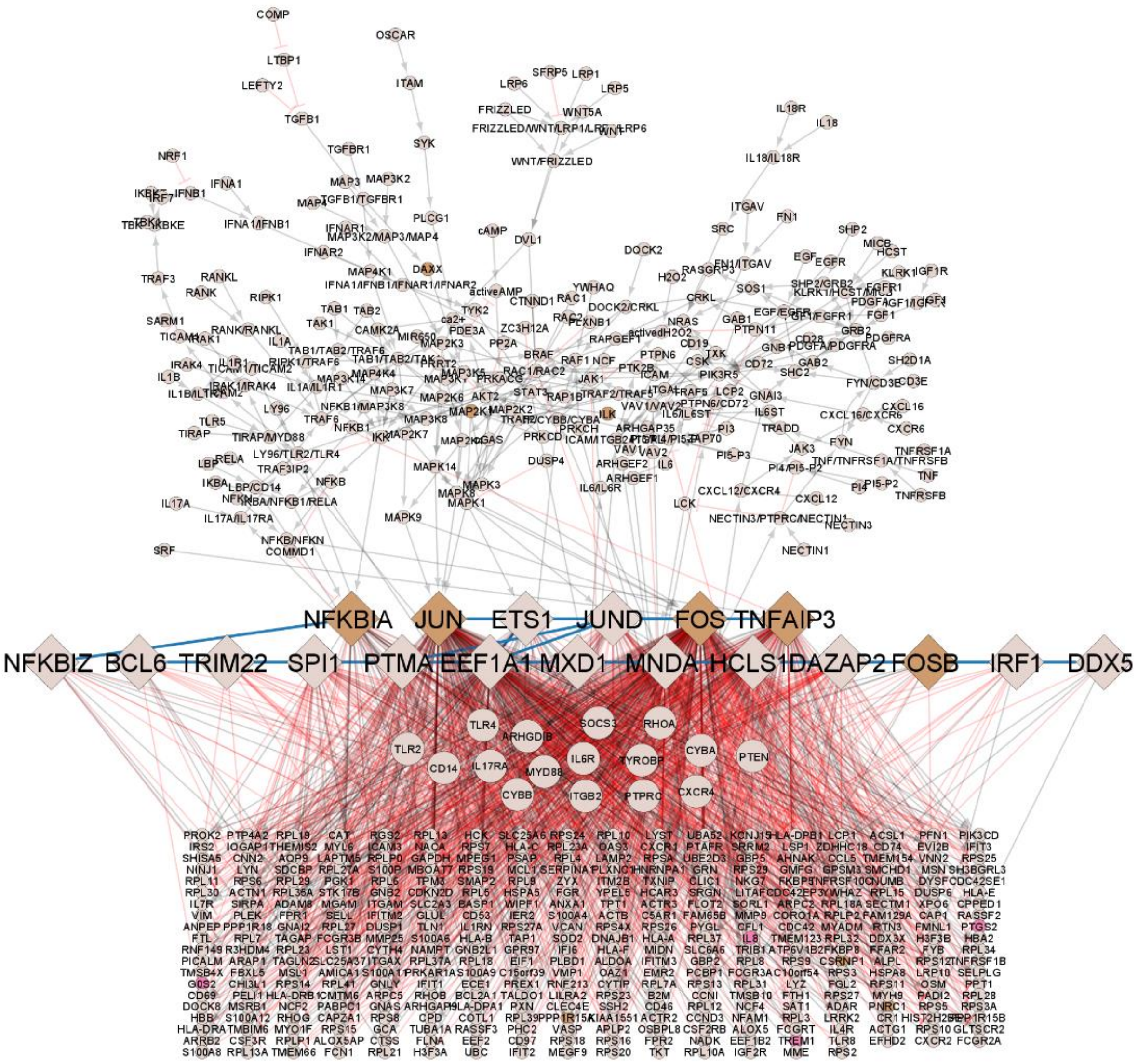

Figure S2. Global RA network and DEG from responders/non-responders to anti-TNF treatment (37 and 41 RA patients treated with Adalimumab and Etanercept, respectively). Overlapping DEG from Adalimumab treatment and Etanercept treatment data are shown in brown (11) and pink (4), respectively, while non-overlapping genes/proteins are shown in grey (599). Transcription factors are depicted in diamond shapes, while upstream regulators and target genes are depicted using round shapes.

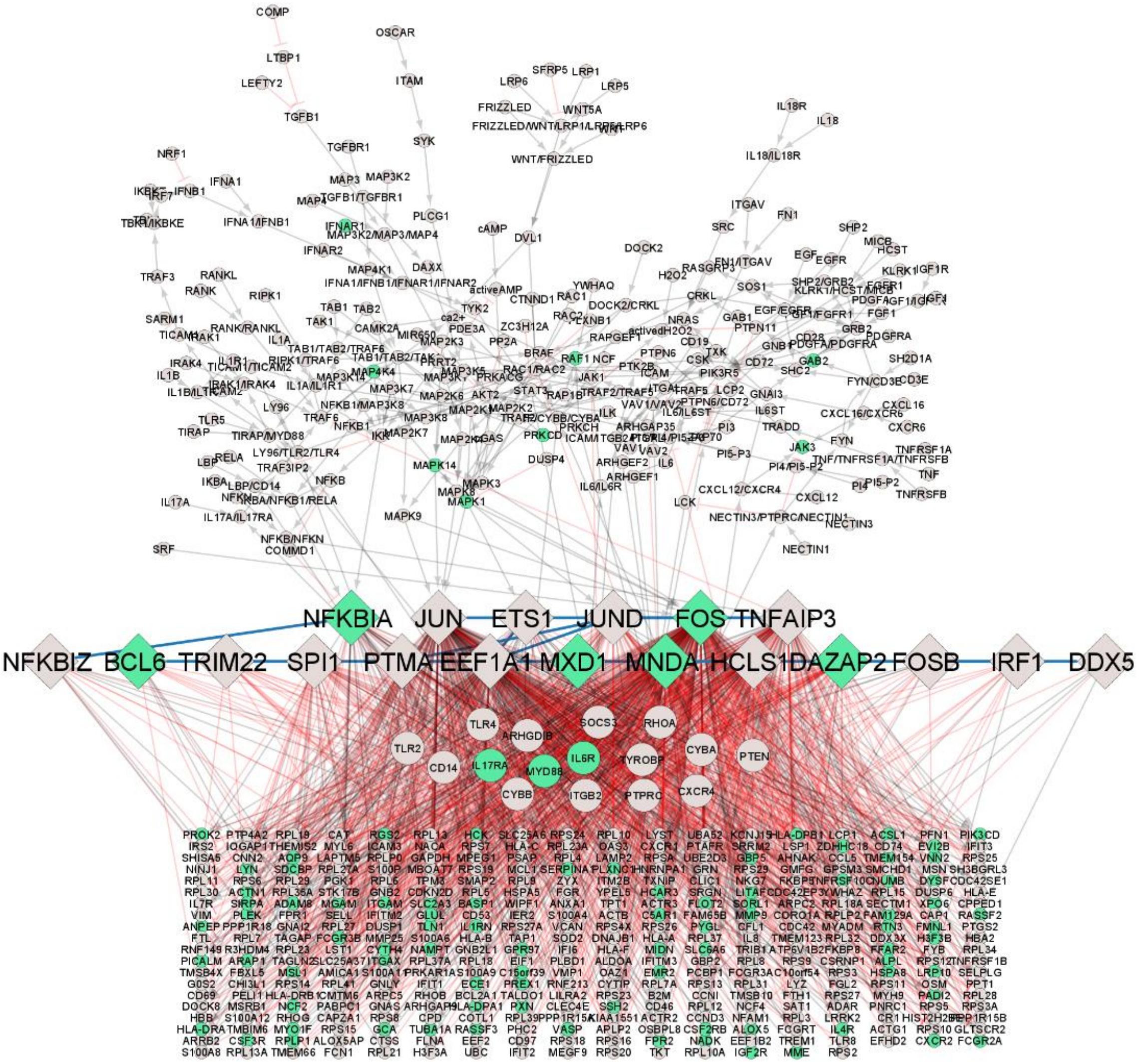

Figure S3. Global RA network and DEG from untreated and treated RA patients with anti-TNF treatment (two different cohorts of 40 and 36 RA patients from baseline and after three months treatment with Infliximab or Adalimumab). Overlapping DEG are shown in green (101), while non-overlapping genes/proteins are shown in grey (513). Transcription factors are depicted in diamond shapes, while upstream regulators and target genes are depicted using round shapes.

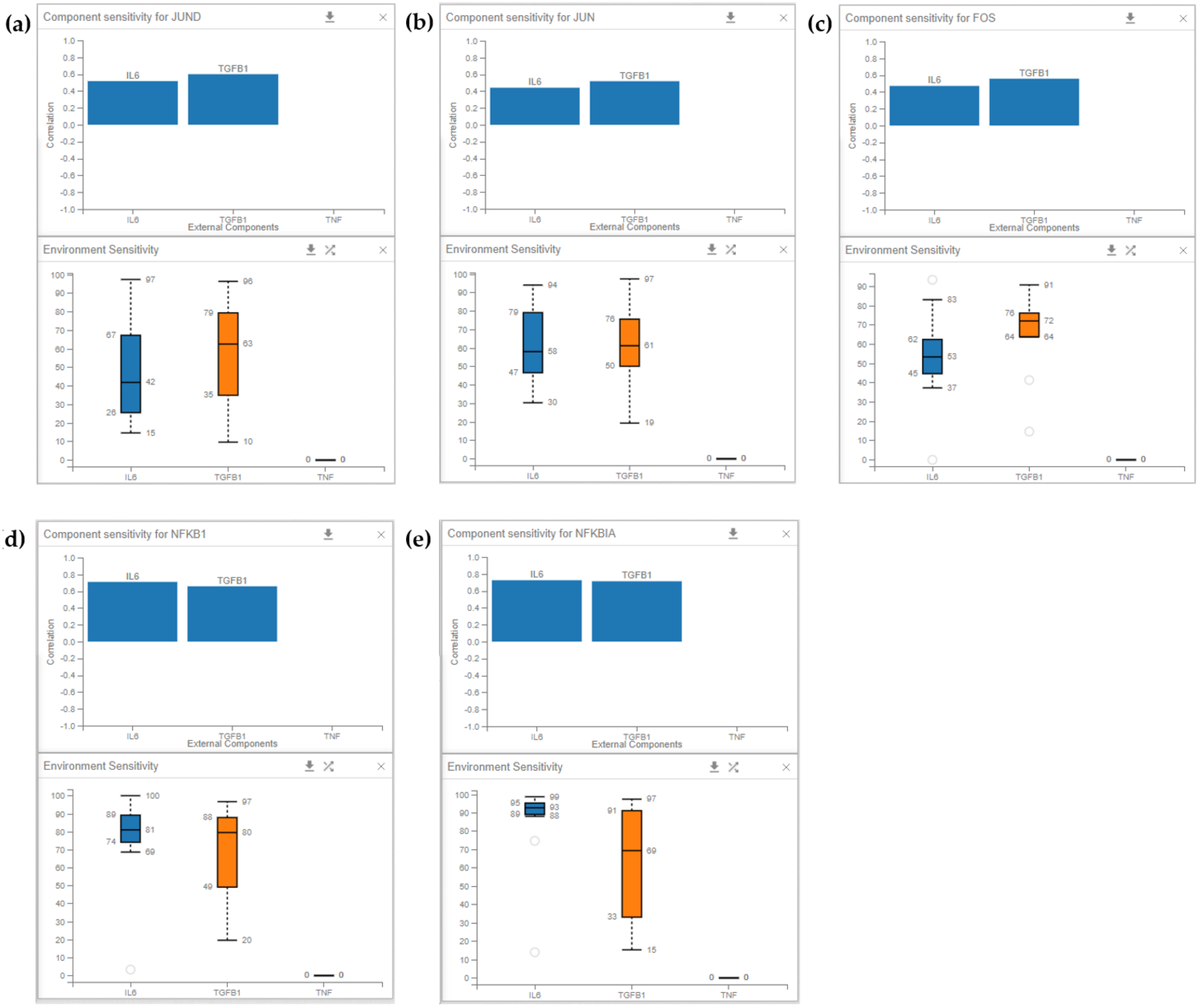

Figure S4. Environment sensitivity analysis. Transcription factors (TFs) could be upregulated in the absence of TNF activity for a combination of activity ranges of the other two inputs (IL6 and TGFB1). The upper part of each subfigure shows the impact of the external components on the activity state of the selected TFs, and the lower part of the image shows the range of activity percentage of the external components to achieve the optimisation of the activity state of the selected TFs. Sensitivity analysis for (a) JUND; (b) JUN; (c) FOS; (d) NFKB1; (e) NFKBIA; boxplots of IL6 in blue, TGFB1 in orange and TNF as black line as it is set to off.

Table S1. Transcription factors identified from CoRegNet and their involvement in RA based on literature evidence.

No	Transcription factors	Role in RA	References
1	**TNFAIP3**	NF-κB target gene, also involved in negative-feedback mechanism to block NF-κB activation through its ubiquitin-editing function in response to various inflammatory signaling, including TNF, IL-1β	PMID: 20822710<br>PMID: 22402800<br>PMID: 20852893<br>PMID: 26405544
2	**IRF1**	IRF1 is critical for the TNF-driven interferon response in rheumatoid fibroblast-like synoviocytes	PMID: 31285419<br>PMID: 21834067<br>PMID: 32765497
3	**ETS1**	Factor involved in the cytokine-mediated inflammatory and destructive cascade which is a characteristic of RA	PMID: 23101665<br>PMID: 11229456<br>PMID: 11976735
4	**FOS**	Subunit of AP1 transcription factor which is involved in the transcriptional regulation of many pro inflammatory genes in RA	PMID: 19395871<br>PMID: 8660103<br>PMID: 9153554
5	**NFKBIA**	Involved in different pathways and cellular processes such as TNFα signalling via NFκB	PMID: 30468518<br>PMID: 18454843
6	**JUND**	Subunit of AP1 transcription factor which is involved in the transcriptional regulation of many pro inflammatory genes	PMID: 9764613<br>PMID: 17515956

**7**	**HCLS1**	Dysregulated in RA synovial tissue	PMID: 12905466<br><br>PMID: 19563633
**8**	**SPI1**	Essential for the expression of gliostatin/thymidine phosphorylase in RA which has angiogenic and arthritogenic activities	PMID: 22534375<br><br>PMID: 28192374
**9**	**MXD1**	Expressed in RA peripheral blood cells, RA synovium	PMID: 22753658<br><br>PMID: 10568429
**10**	**JUN**	Subunit of AP1 transcription factor which is involved in the transcriptional regulation of many pro inflammatory genes in RA	PMID: 18454843
**11**	**NFKBIZ**	Involved in TNF and IL-17 mediated signaling	PMID: 32079724
**12**	**TRIM22**	Expressed in RA peripheral blood	PMID: 24756903
**13**	**FOSB**	Subunit of AP1 transcription factor which is involved in the transcriptional regulation of many pro inflammatory genes in RA	PMID: 29326694
**14**	**DDX5**	DDX5 is required for the transcription of key Th17 genes involved in Th17-mediated autoimmune inflammation in RA	PMID: 29254845
**15**	**BCL6**	Interleukin-29 regulates T follicular helper cells by repressing BCL6 in RA	PMID: 16508929<br><br>PMID: 28150777<br><br>PMID: 32468318

**16**	**MNDA**	Citrullinated protein identified in RA synovial fluid; Interferon induced nuclear and cytoplasmic protein	PMID: 23044660<br><br>PMID: 15158620
**17**	**EEF1A1**	Expressed in RA peripheral blood	PMID: 21444302
**18**	**PTMA**	Regulated by c-Myc, an oncoprotein overexpressed in synovium of RA, and is associated with cell proliferation	PMID: 17372028 (mice)
**19**	**DAZAP2**	Expressed in RA peripheral blood mononuclear cells (PBMCs)	PMID:26352601

List of abbreviations

AF: Activity Flow
BCL6: BCL6 Transcription Repressor
CaSQ: CellDesigner as SBML-Qual
ChIP: Chromatin Immunoprecipitation
DAZAP2: DAZ Associated Protein 2
DAXX: Death Domain Associated Protein
DEA: Differential Expression Analysis
DMARDs: Disease-Modifying Anti-Rheumatic Drugs
ETS1: ETS Proto-Oncogene 1, Transcription Factor
EEF1A1: Eukaryotic Translation Elongation Factor 1 Alpha 1
EI: Evidence Index
FDR: False Discovery Rate
FOS: Fos Proto-Oncogene, AP-1 Transcription Factor Subunit
FOSB: FosB Poto-Oncogene, AP-1 Transcription Factor Subunit
HCLS1: Hematopoietic Cell-specific Lyn Substrate 1
IRF1: Interferon Regulatory Factor 1
ILK: Integrin Linked Kinase
IL6: Interleukin 6
JUN: Jun proto-oncogene, AP-1 transcription factor subunit
JUND: JunD Proto-Oncogene, AP-1 Transcription Factor Subunit
KO: Knock Out
MXD1: MAX Dimerization Protein 1
MAPK1: Mitogen-Activated Protein Kinase 1
MAPK14: Mitogen-Activated Protein Kinase 14
MAP2K1: Mitogen-Activated Protein Kinase Kinase 1
mAbs: Monoclonal Antibodies
MNDA: Myeloid Cell Nuclear Differentiation Antigen
MTX: Methotrexate
NFKBIA: NFKB Inhibitor Alpha
NFKB1: Nuclear Factor Kappa B Subunit 1
PCA: Principal Component Analysis
PTMA: Prothymosin Alpha
RA: Rheumatoid Arthritis
SIF: Simple Interaction Format
TCZ: Tocilizumab
TNFAIP3: TNF Alpha Induced Protein 3
TF: Transcription Factor
TGFB1: Transforming Growth Factor Beta 1
TRIMM22: Tripartite Motif Containing 22
TNF: Tumor Necrosis Factor
VDA: Variant Disease Association
